# High prevalence of minor neurologic deficits in a long-term neurodevelopmental follow-up of children with severe persistent pulmonary hypertension of the newborn: a cohort study

**DOI:** 10.1186/1824-7288-36-45

**Published:** 2010-06-13

**Authors:** Anna Berti, Augusta Janes, Riccardo Furlan, Francesco Macagno

**Affiliations:** 1 Neonatology Division, AO S. Chiara, Trento, Italy; 2 Neonatology Division, AOU S. Maria della Misericordia, Udine, Italy

## Abstract

**Background:**

Persistent pulmonary hypertension of the newborn (PPHN) is a severe condition that determines a profound brain hypoxia. Inhaled nitric oxide was approved for the treatment of PPHN since the end of the 1990s. The debate upon the long term outcome of these children is still open. Our aim was to investigate the incidence of minor long-term neurodevelopmental problems in a cohort of children affected by severe PPHN.

**Methods:**

All neonates with severe PPHN treated with inhaled nitric oxide in our facility between 01.01.02 and 31.12.07 were seen in a follow up visit and evaluated with a neurodevelopmental scale, according to their age at the time of observation.

**Results:**

in the study period 31 children were diagnosed with severe PPHN. 29 survived. 27 accepted to come for follow-up. Mean age: 41 months (range 12 - 70 months).

26% of the evaluated children had some behavioural problems, while 22% had some language disturbances.

**Conclusions:**

This is the first neurodevelopmental follow-up of neonates with PPHN in which children older than 36 months have been evaluated.

There is an unexpected high incidence of minor neurological deficits, mainly regarding the fields of language and behaviour. These deficits seem to be related to the severity of illness rather than to the treatment. Language and behaviour are considered "higher functions" in humans and their integrity can be better defined in older children.

## Background

Persistent pulmonary hypertension of the newborn (PPHN) is a clinical syndrome that results from failure of the pulmonary circulation to adapt to extrauterine life[1-3].

There is a persistently elevated pulmonary vascular resistance, associated with a right-to-left shunting through the foramen ovale and/or ductus arteriosus.

Although PPHN is commonly associated with a parenchymal lung disease, about 20% of the cases are classified as idiopathic[4,5].

Hypoxia is one of the major causes of PPHN.

The clinical presentation of PPHN might be the result of different pathophysiological processes, but the diagnosis is anyway based on three fundamental features:

- severe hypoxia

- right-to-left shunting, proven by echocardiography

- absence of a cyanotic congenital heart disease

PPHN is to be suspected in case of very poor oxygenation and particularly when hypoxia is relatively too deep compared to the severity of pulmonary parenchymal disease or to the effectiveness of CO_2 _exchanges.

The severe instability of these patients make them susceptible to respiratory and cardiac complications but also to neurological sequelae, considering the effects of pressure impairment on cerebral blood flow and the immaturity and fragility of the newborn's central nervous system.

To our knowledge, relatively few studies have tried to evaluate the long-term neurodevelopmental outcome of newborns affected by PPHN, often leading to discordant results. Previous works reported a 10% to 20% rate of long-term neurodevelopmental sequelae in infants with PPHN[6-9].

We know that neonates who needed prolonged mechanical ventilation are at risk for minor neurological deficits: hypo-oxygenation as a matter of fact plays a relevant role in the pathogenesis of brain damage, especially in terms of cortical organization, associative cortical areas or neurotransmitters system. This condition affects the normal development of intellectual skills and behaviour, so that cognitive outcome could be particularly compromised in case of severe neonatal hypoxia.

Our study had two main objectives:

1. To carry out a long-term neurodevelopmental follow-up of the children with PPHN treated with nitric oxide in the Neonatology Division of Udine (Northern Italy) over a period of 6 years.

2. To revise retrospectively these cases considering enrolment criteria to treatment with nitric oxide (NO), nitric oxide dosage, clinical response and short-term outcome.

## Patients and methods

### Patient population

From the 1^st ^of January 2002 to the 31^st ^of December 2007, 31 neonates were admitted in the Neonatal Intensive Care Unit of The S. Maria della Misericordia Hospital in Udine with a diagnosis of severe PPHN confirmed by echocardiographic findings and were treated with inhaled nitric oxide (iNO).

We retrospectively selected from the database all children with a proven diagnosis of PPHN with echocardiographic evidence of pulmonary hypertension who had been treated with iNO. Patients who did not undergo echocardiography were excluded, as were those who did not receive iNO. The excluded patients are not reported in this study.

Incidence of PPHN was about 1:1000 born alive.

Data recorded from the neonatal period included sex, gestational age, weight, APGAR score, admitting diagnosis. An *ad hoc *database was created to retrospectively collect all these data and analyze them. We also analyzed ventilation parameters such as mean airways pressure (MAP), inspired oxygen fraction (FiO_2_), haemogas-analysis values (pH, PaO_2_, PaCO_2_), oxygenation index (OI), alveolar-arterial oxygen gradient (AaDO_2_) and arterial blood pressure values.

All these parameters were measured before starting iNO therapy, at any significant variation of mechanical ventilation or any relevant change in clinical conditions, until discontinuation of iNO and/or resolution of PPHN.

### Clinical management

All enrolled children presented severe hypoxic respiratory failure and were treated with conventional mechanical ventilation or high frequency oscillatory ventilation (HFOV, SensorMedics model 3100A).

Inotropic agents (dopamine and/or dobutamine) were administered for blood pressure support and sedation with fentanyl was performed too[10-12].

Patients were eligible for iNO therapy if they had PPHN clinical features and there was echocardiographic evidence of indirect signs of pulmonary hypertension.

In our facility, we usually perform cardiac ultrasound before starting iNO because we can perform it by ourselves in the NICU. In the 31 considered patients, cardiac ultrasound was always performed before starting iNO.

An initial dose of 20 ppm iNO was used, according to previous Italian and international trials[11,13-21].

Responses to iNO were classified into two groups: early response was defined as a 25% OI reduction occurring within 6 hours after iNO initiation; late response as OI reduction within 6-24 hours. Infants who did not show a 25% OI reduction after 24 hours were considered unresponsive[15-17,22].

### Follow-up programme

All infants who survived were offered participation in our study and they were invited to come for a clinical and neurodevelopmental evaluation.

Informed consent was given by parents of each infant.

Some of the inborn patients had also been seen before in our conventional follow-up programme (every third month).

Outborn patients were supposed to have been seen in the centre closest to their home, but some of them had had no follow-up at all.

In the follow-up visit, we interviewed parents on growth and development, intercurrent illnesses, respiratory problems, rehospitalization.

Growth parameters were measured: weight, height and cranial circumference were plotted on the reference curves[23]. A complete physical examination was performed.

Neurodevelopmental examination was based on Bayley Scales of Infant Development[24] for children 12 to 42 months of age. Standards developmental scores are available: Standards Scores Percentiles Developmental Age Equivalents. The lower limit for the Mental Developmental Index (MDI) is considered 50 and results can be included in four descriptive classifications: accelerated performance, normal range, mild delay, severe delay.

Older patients were tested with Wechsler Preschool and Primary Scale of Intelligence WPPSI[25].

The final scores are classified as extremely low if they are < 70; borderline between 70 and 89; in the average range between 90 and 109; higher scores are considered superior or very superior (> 130). One child with cerebral palsy was tested using the Griffith score[26].

Both for Bayley Scales and WPPSI, the scores of the mental/verbal area (MDI) and those concerning the performance/motor area (PDI) were considered separately, so that total score was not falsely decreased by a specific deficit in a single area.

### Data analysis

Data analysis was performed in the Epidemiology Institute of the University of Udine. Comparison analyses were carried out with the SAS software.

Frequencies of the neurodevelopmental scores, underlying diseases, gestational age and of any ventilation parameter were calculated.

Some variables have been categorized.

Statistical significance of the differences between groups was evaluated through chi-square (χ^2^) analysis and Fisher test.

Our sample population is not very numerous and this may decrease the statistical significance of our results. Anyway PPHN is a relatively rare condition and only multicentric studies can present larger samples.

## Results

31 children met the criteria for the diagnosis of PPHN.

Underlying conditions included: meconium aspiration (7/31), pneumothorax (5/31), severe asphyxia (5/31), respiratory distress syndrome (3/31), sepsis (3/31), adenomatoid cystic pulmonary malformation (2/31), placental disruption (2/31), idiopathic PPHN (4/31).

The minor cardiovascular defects that we found were not directly responsible for the clinical manifestations of PPHN. They included only small defects of interventricular and interatrial septum and irrelevant patent ductus arteriosus. None of these was hemodinamically significant. In these cases PPHN was considered idiopathic, given that there was not another major cause.

Major congenital cyanotic heart diseases were excluded from the study.

We had no cases of diaphragmatic hernia.

Male sex was sharply prevalent: 25/31 were male (80.6%); 6/31 female (19.4%).

Median gestational age was 38 weeks (range 26 - 41 weeks).

The range of gestational age seems to be wide, but really premature children were very few; actually only 5 children were born at less than 34 weeks. One was 26 weeks and one was 27 weeks; the other three were older than 30 weeks. None of them showed any major complication of prematurity (including IVH, ROP or BPD) that could be responsible for the long-term outcome. All of them had negative cerebral ultrasound and none of them had any major neurological deficit.

Median weight at birth was 3.200 gr (range 1.100 - 4.640 gr) and 29 out of 31 newborns had an adequate for gestational age weight (AGA); the two remaining children were considered large for gestational age with weight > 97° percentile.

APGAR score was < 6 at 5 minutes of life in 6 cases.

All infants were treated with iNO. Nitric oxide administration was started at the post-natal mean age of 1.4 days, according to literature[14,16,27,28]. The median initial dose of iNO was 20 ppm (range 15 - 30); the maximum dosage of 40 ppm, recommended by Italian studies for unresponsive patients[15], was achieved just in one case. A dose of 80 ppm, reported in some International trials[18,29-31], was never reached.

16 patients (51.6%) of the studying population received conventional mechanical ventilation; the remaining 15/31 received high frequency oscillatory ventilation (HFOV). In our facility, we usually started HFOV in the patients in whom PPHN was associated to a particularly severe parenchymal pulmonary disease.

Average initial MAP was 11.8 cmH_2_O (range 5 - 23).

FiO_2 _was 100% in all cases and we attempted optimization of ventilation before starting iNO treatment. Mean oxygenation index was 28.36, while median OI was 27 (range 76.5 - 5).

15 infants (48.4%) showed an early response to treatment, while 14 (45.2%) had a late response. 2 neonates (6.4%) were unresponsive and their oxygenation index worsened even after starting inhaled nitric oxide.

No statistical significant differences were found between early and late responders in term of initial OI, type of ventilation, duration of iNO treatment, iNO dosage or any other recorded parameter.

Response was not even statistically dependent on gestational age or underlying pathology. Table 1.

**Table 1:** Summary of results in the two groups of early and late responders.

	Early response (n = 15 48.38%)	Late or no response (n = 16 51.62%)
Male sex	11	14
Mean gestational age	35.4	37.2
Outborn	9	12
Urgent cesarean section	9	7
Age at iNO initiation (hours)	27.5	37.9
Total iNO hours	74.5	84.5
Maximum iNO dosage (ppm)	23.3	26.1
Maximum OI	33.7	23.3
Underlying condition		
Primary PPHN	0	4
Meconium aspiration syndrome	4	3
RDS	3	0
Asphyxia	2	1
Pneumonia	2	2
PNX	4	2

2 infants died before discharge (6.4%), but their death was not directly related to PPHN itself: one child was severely compromised by septic shock; the other one was affected by congenital haemochromatosis and presented a multi-organ failure confirmed by post-mortem examination.

29 children survived, yet only 27 accepted to come for follow-up.

At the follow up visit, auxologic parameters were included between 10° and 90° percentile for all children.

No significant respiratory pathologies were found.

Mean age at the time of the neurodevelopmental evaluation was 41 months (range 12 - 70). This is the first follow-up study of children with PPHN in children older than 36 months.

The wide age range at the time of observation is due to the long period considered for the study (from 01.01.02 to 31.12.07). Anyway, only nine patients were younger than 36 months at the time of neurodevelopmental examination, while 18 children out of 31 were 36 months or older.

14/27 children (51.8%) have been evaluated with the Bayley Scales[24] and their average age was 26 months (range 12 - 42). 12/27 children (44.5%) did the WPPSI[25] test at an average age of 60 months (range 46 - 70). One child underwent the Griffith[26] test as he was affected by severe cerebral palsy with spastic tetraplegy and mental retardation. For this child and for another one in the WPPSI group, it was not possible to determine a numeric score. Therefore we have a numeric score just for 25 patients.

We found a pathologic score (< 70) in the mental/verbal area in 5 out of 25 cases (20%).

The incidence of a performance/motor score < 70 is 8%. Table 2.

**Table 2:** Numeric scores resulting from Neurodevelopmental Tests.

	Scores	< 70	70-85	86-115	> 115
**BAYLEY (n = 14)**	MDI	2 (14%)	2 (14%)	7 (50%)	3 (22%)
	
	PDI	0	3 (22%)	9 (64%)	2 (14%)

**WPPSI (n = 11)**	Verbal	3 (27%)	2 (18%)	5 (46%)	1 (9%)
	
	Perform.	2 (18%)	1 (9%)	7 (64%)	1 (9%)

**Total (n = 25)**	Verbal /MDI		4 (16%)	12 (48%)	4 (16%)
	
	Perform./PDI		4 (16%)	16 (64%)	3 (12%)

Despite the numeric score, in the complete sample of 27, 6 children turned out to have deficits in the linguistic area (22%).

7 (26%) patients, moreover, showed "minor" behavioural problems even tough they did not have a pathologic MDI score.

Electrophysiological tests have been performed in 17 out of 25 of our children, but none of them showed significant abnormalities.

All children underwent cerebral ultrasound in the neonatal period. It turned out negative in 22 cases; mild persistent flare was reported in 7 cases. Only two children had IVH of III° or IV° grade: one of these is the one with cerebral palsy (CP); the other is one of the two who died before discharge.

7 cases underwent a cerebral magnetic resonance, but it resulted negative except for the child with CP.

Statistical analysis could not prove any significant association between the neurodevelopmental score and the underlying pathology determining PPHN. The neurodevelopmental outcome does not statistically correlate with the early or late response to iNO treatment.

## Discussion

In the present medical setting, treating pathologies and healing the single patient is not any longer enough.

We need to verify the effectiveness of our practice.

The result to be evaluated is not only the short-term outcome and the survival of our patients, but also the long-term impact on population and quality of life.

Actually, we found an excellent short-term outcome.

The raw mortality rate is 6.4% (2/31). It seems to be lower than what presented in literature[28,32-39], yet we cannot directly compare our mortality rate with those reported in other studies, as many other variables may affect them.

In the current clinical practice in our NICU, the criteria to start iNO treatment are based on clinical conditions of the patients with severe hypoxemia, unresponsive to mechanical ventilation with inspired oxygen fraction of 100% and with echocardiographic evidence of PPHN.

OI was calculated ex-post just for this study. So we found that iNO was given to children with a very wide range of OI (5 - 76.5) and 17 cases had an OI lower than 25, which is the accepted cut-off for iNO initiation[10,12,14,16,17,40].

Even tough an earlier initiation of iNO is not associated with lower mortality, it might be related to a minor rate of illness progression to more severe stages[19,39].

As we consider long-term outcome, the great majority of works on children with PPHN try to demonstrate whether there is a difference in the incidence of neurological deficits in those treated with iNO as opposed to those who received other therapies[7,28,35-38,40-43].

The Nitric Oxide Study Group in 2000 published data[28] that did not underline any differences in the neurodevelopmental outcome between children who had received iNO and those who had not received it. Davidson[18] also found similar results.

Minor disabilities were not evaluated in either of these studies.

In his follow-up, Lipkin[35] analyzed minor deficits and concluded that PPHN itself involves a higher risk of neurological sequelae compared to general population, notwithstanding the type of treatment.

Haemodynamic instability during PPHN might cause pressure and metabolic impairment that may alter the auto-regulation of the cerebral blood flow of the newborn[12,44].

In our study, we start from the hypothesis that PPHN is a severe pathology and infants who underwent prolonged invasive ventilation should have a neurodevelopmental follow-up aiming especially at the search for minor deficits[6].

15 of the 27 children (56%) who accepted to come to our follow-up had not had any previous evaluation of the psychomotor development. This might be related to the good general conditions of these patients after discharge from NICU, but also to the fact that many of them (67.7%) came from areas that refer to other hospitals.

In literature, the reported incidence of neurodevelopmental deficits in infants with PPHN is 10% to 20%[6-8]. We found a mental/verbal score < 70 in 20% of our sample, but we also found 22% with language deficits and 26% with behavioural problems.

The incidence of language disturbances seems to be very high and they are more evident in the group of children who were tested with the WPPSI test.

Published data are based almost exclusively on the Bayley Scales performed on children with a mean age of 20-24 months.

At this age language evaluation is limited and a language delay or alteration might be evident only later on. Our study is the first neurodevelopmental follow-up for infants with PPHN, in which children older than 36 months have been evaluated. We found both expressive and receptive linguistic deficits, that did not seem to be related to any auditory impairment.

Neuroradiological examination did not show any major abnormality of the cerebral parenchyma, and cerebral ultrasound that had been performed in all children in the neonatal period was normal in the children who had language or behavioural problems at follow up visits.

In human beings, language is a higher function and not just a motor or sensitive function regulated by a single functional brain area.

Studies on post-traumatic aphasia demonstrate that there is no direct relation between lesions in the classical linguistic areas (Broca and Wernike) and the development of the corresponding forms of aphasia[45].

The ability to comprehend and produce language is based on an interwoven constellation of skills influenced by exogenous factors (social, physical and linguistic interactions with the environment) and by endogenous factors such as the interactions among neural systems. The anatomo-functional subdivision of cortical areas then does not follow established developmental trajectories, but is driven by the sensimotor experience of the individual[45].

PPHN can cause a diffuse and multifocal hypoxic brain damage that does not determine the development of major neurological deficits such as cerebral palsy, but it might involve and compromise the connection network among different cortical areas.

This kind of damage may involve more subtle clinical features that become evident in older children who are required more elaborated performances, such as linguistic expression or the maintenance of attention while performing a specific task.

A correct follow-up programme is then important to allow early identification of these problems. Moreover, it might prevent a negative evolution by starting a specific treatment as soon as possible.

The therapeutic alliance with the family established in the follow-up programme may also be helpful to limit or correct behavioural disturbances that were proven to be relevant in these children.

Follow-up must continue to pre-school and school age, so that specific deficits regarding language, calculation skills and/or abstract thinking may be found out.

## Conclusions

We already knew that PPHN is a very severe condition associated with a high mortality rate, but also with the risk of long-term neurodevelopmental sequelae. We also knew that inhaled nitric oxide is an effective treatment to reduce mortality and the need for ECMO.

Thanks to the results achieved through this study, we can say that it might be reasonable to start iNO therapy on the base of clinical criteria even in children with OI < 25. This attitude might not reduce total mortality rate, but it could be associated with a lower rate of illness' severity progression and thus with a better long-term outcome.

Anyway, specific trials to evaluate this topic are not yet available.

Regarding long-term outcome:

- we did not find a significant incidence of respiratory problems;

- neurodevelopmental tests showed a high prevalence of minor deficits, especially in the linguistic area. These children are asymptomatic for major neurologic disabilities and so they can be lost in normal follow-up programmes;

- children with PPHN are exposed to hypoxemia and therefore they need to be monitored in a strict follow-up programme for psychomotor development, possibly up to school age.

Having a better knowledge of the long-term neurodevelopmental deficits in children who were admitted in NICU, we get an improved awareness on the effects of pathologies and their treatment.

Up to now, more researches are certainly needed to better comprehend the neuro-physiological alterations underlying the minor deficits of these children.

## List of abbreviations

PPHN: Persistent pulmonary hypertension of the newborn; NO (iNO): Nitric oxide (inhaled); RDS: Respiratory distress syndrome; PaO2: Arterial partial oxygen pressure; PaCO2: Arterial partial CO2 pressure; SatO2: arterial oxygen saturation; HFOV: High frequency oscillatory ventilation; FiO2: Inspired oxygen fraction; MAP: Mean airway pressure; ECMO: Extracorporeal membrane oxygenation; AaDO2: Alveolar-arterial oxygen gradient; OI: Oxygenation index; NINOS: Neonatal inhaled nitric oxide study group; AGA: Adequate for gestational age; MDI: Motor developmental index; PDI: Performance developmental index; NICU: Neonatal intensive care unit.

## Competing interests

The authors declare that they have no competing interests.

## Ethical approval

Not required. This is a retrospective study. All neonates had been treated according to the therapeutic protocol for PPHN already approved by our Ethical Committee. Informed consent for neurodevelopmental evaluation was given by parents of each child.

## Authors' contributions

AB, AJ, RF and FM designed the study protocol; AB, AJ and RF were directly involved in the clinical management of the patients. AB, AJ and RF carried out the data collection and carried out the analysis and interpretation of the data. AB, AJ and FM drafted the article. All authors read and approved the final manuscript. AB and AJ are guarantors of the paper.

## Funding

None.
